# The Scheme of the British Medical Association.—II

**Published:** 1918-05-25

**Authors:** C. Thackray Parsons


### A MINISTRY OF HEALTH.

### The Scheme of the British Medical Association.—II.

By C. THACKRAY PARSONS, M.D.Lond.

Most medical men agree that a Ministry of Health is desirable. They find their work hampered every day by the present haphazard methods of dealing with sickness in the individual and the community. Any scheme tending to simplify and co-ordinate the provision made by the public to improve the health of the nation is certain of their sympathetic attention. Any criticism will be directed solely to an examination of the details of the changes proposed. A consideration of the scheme summarised in our issue of May 18 shows that the British Medical Association has kept in view six principles:—(1) The equal importance of the clinical and preventive services; (2) the maintenance of local administrative bodies with full powers in all matters of health administration within their areas, subject to the same control by the Ministry of Health as is now exercised by the various central departments to be absorbed; (3) the preservation of voluntary hospitals, supplemented by State hospitals; (4) the utilisation of every medical practitioner willing to take service under the scheme; (5) the linking up of research work with the Ministry of Health; and (6) the importance of co-ordinating the public provision for lunatics and mental defectives with the Ministry of Health, so as to discourage the idea that there is any difference in kind between mental and bodily disease. No one of these principles, with the possible exception of the last, would meet with the approval of all medical men, but probably a majority would agree with them. It is when the details of their application in practice are laid down that disagreement chiefly arises. It will be worth while, therefore, to consider under each heading how the British Medical Association proposes to apply them.

### 1. The Equal Importance of the Clinical and Preventive Services.

There was at one time a tendency amongst members of the Public Health Service to take up the position that all public medical work should be organised by their Service—a tendency to which the Minority Report of the Royal Commission on the Poor Laws gave great impetus. There is now general agreement that this position is untenable. Under the Association scheme the two Services are to be on an equal footing and are to be co-ordinated by a chief administrative medical officer in each area—the man with the best qualifications for the office to be selected in whatever branch of the Service he may have worked. There is no doubt that these suggestions will meet with general approval.

### 2. The Maintenance of Local Administrative Bodies.

One school of medical men is in favour of bringing the administration of public medical work throughout the country under the direct control of the Ministry of Health, the necessary funds to be obtained from the State, from the contributions of the insured, and from a universal national health-rate collected by the local authorities. There is much to be said for this view, but it is such a complete revolution of the present system that it is hardly likely to be accepted by the general public. The scheme of the Association will also meet with much opposition. Local patriotism makes the carving of the country into the most suitable areas for health administration a very difficult task. Still, much could be done in this direction without exciting strong resistance. The proposed composition of the Local Health Committee is open to much greater objection. Full details are not given, but most of its members would apparently be non-elected. Most people will be of opinion that a body having the large powers that are to be entrusted to it should be subject, as regards the majority of its members, to public election.

### 3. The Preservation of Voluntary Hospitals.

Although many medical men think that the difficulty of maintaining voluntary hospitals will soon become so great that State hospitals will gradually replace them, the majority would wish them preserved. Under the scheme of the Association they would be preserved, but they would become more and more State-aided institutions. This State aid is accompanied by conditions. The members of the staff are to be paid for their attendance on patients under the public scheme, and the practice of the hospital is to be open to all medical students and practitioners. Any increase in State aid would probably be accompanied by further interference with the administration of the hospital, until there would be little difference between the State-aided and State-maintained hospital. If the voluntary hospital is to retain its distinctive characters the amount of State aid it receives must be a small proportion of its income. No indication is given as to what patients entitled to treatment under the public scheme would be sent to voluntary hospitals and what to the State hospitals. It is important that the selection should be so made that it would not be possible for the public to get the idea that the one kind of hospital was in any way inferior or less desirable than the other.

### 4. The Utilisation of the Medical Practitioner.

There is a great difference of opinion amongst medical men as to what would be the best form of relation between the Ministry of Health and the general body of medical practitioners. There is the view of the Association that the number of whole-time salaried medical officials of the Ministry should be kept as low as possible, the bulk of the work

☛ The previous article appeared on May 18, p. 138.

**A MINISTRY OF HEALTH**—*(continued)*.

being done by medical men in private practice; there is the view that the whole profession should form a salaried public Service, private practice being reduced to a very small amount; and there is the view that there should be a whole-time Service consisting of medical officers of health, institutional medical officers, and medical officers for the domiciliary treatment of the non-insured poor, the rest of the public work being done by private practitioners. The scheme of the Association would work well in rural districts where small institutions of the cottage hospital type would meet all the ordinary needs of the population. It would be impracticable in the case of large institutions serving thickly populated areas. In such an institution the administrative difficulties in the way of allowing all the practitioners in the area to attend the cases sent in by them would be insurmountable. In the case of London and the larger county boroughs it would be advantageous to have whole-time officers even for the specialist appointments. There should, however, be close co-operation between the hospital staff and the medical men in the district to ensure continuity of treatment and the use of the hospital to the greatest advantage. This could be arranged by giving each medical man a consulting-room at the hospital. Every patient, on his discharge, could then be seen by the medical man, who would be responsible for any subsequent treatment required, and there would be easy means of consultation between the indoor and outdoor staff on every case recommended for admission.

5. THE LINKING-UP OF RESEARCH WORK.

The only objection to this is the fear that research might not flourish in an official atmosphere. The useful work done by the Research Department of the Local Government Board should allay that fear. The wealth of material at the disposal of a Ministry of Health would be so great that a department for research in connection with it must be formed.

The only other important point for criticism in the Association scheme is the proposed Advisory Council of the Ministry. The composition of the Council is not clearly stated. The medical profession is to have representatives, but the other interests with claims to representation are nowhere specified. Great stress is laid upon the importance of giving this Council power to report direct to Parliament if the Minister acts against its advice. It is difficult to believe that Parliament would give such power to an Advisory Council. If it did, the position of the Minister would not be a pleasant one.

